# The Assessment of Knowledge About Cervical Cancer, HPV Vaccinations, and Screening Programs Among Women as an Element of Cervical Cancer Prevention in Poland

**DOI:** 10.3390/jpm14121139

**Published:** 2024-12-04

**Authors:** Krystian Wdowiak, Agnieszka Drab, Paulina Filipek, Urszula Religioni

**Affiliations:** 1 Faculty of Medicine, Medical University of Lublin, University Clinical Hospital No. 4 in Lublin, K. Jaczewskiego 8 St., 20-954 Lublin, Poland; krystianrwdowiak@interia.eu; 2 Department of Medical Informatics and Statistics with e-Health Lab, Medical University of Lublin, 20-059 Lublin, Poland; 3 Faculty of Health Sciences, Medical University of Lublin, 20-059 Lublin, Poland; paulina.filipek@umlub.pl; 4 School of Public Health, Centre of Postgraduate Medical Education of Warsaw, 01-826 Warsaw, Poland; urszula.religioni@cmkp.edu.pl

**Keywords:** cervical cancer, public health, prevention

## Abstract

**Introduction**: Cervical cancer is the fourth most commonly diagnosed malignant tumor in women and the fourth leading cause of cancer-related deaths among this population. Since it is asymptomatic in its early stages, preventive screening plays a crucial role in rapid diagnosis. Such screenings are conducted in many countries worldwide, although their popularity varies. Given that nearly all cases of cervical cancer are linked to high-risk human papillomavirus (hrHPV) infection, vaccination against this virus could lead to a significant reduction in cancer incidence. It should be noted that the level of vaccination coverage against hrHPV varies significantly between countries, ranging from a few percent to over 90%. Globally, the vaccination coverage of the target population is estimated at only a few percent. **Methods**: This study was conducted using a proprietary, anonymous online questionnaire comprising 24 questions addressing various aspects of cervical cancer prevention. The newly designed questionnaire comprised 19 primary questions and 5 metric questions. The collected data were subjected to descriptive and statistical analysis. **Results**: The majority of respondents reported regularly participating in cervical cytology screening and gynecological visits. Non-participation in these screenings was primarily reported by younger respondents, not all of whom had indications for undergoing such examinations. Only 14% of the women surveyed had been vaccinated against hrHPV. However, it should be noted that, as the surveyed women were not covered by the relatively recently introduced vaccination program, they received their vaccinations through local programs conducted by certain cities or through private healthcare services. The respondents’ primary sources of information on cervical cancer are the internet and medical personnel. **Conclusions**: The level of knowledge among the women surveyed regarding cervical cancer prevention was satisfactory, though improvement is needed in some areas. Despite relatively good awareness of HPV’s role in cervical cancer development, the popularity of HPV vaccination remains unsatisfactory. The results should be interpreted with caution due to the small study group.

## 1. Introduction

Cervical cancer is globally the fourth most commonly diagnosed malignant tumor in women (approximately 6.8% of all diagnoses) and the eighth most commonly diagnosed malignant tumor overall (approximately 3.3% of all diagnoses) [1,2,3]. This cancer is also the fourth leading cause of cancer-related deaths among women globally (about 8.1% of all such deaths) and the ninth leading cause of cancer-related deaths overall (approximately 3.6% of all cancer-related deaths) [1,2,3]. It is estimated that in 2022, there were approximately 660,000 new cases and 350,000 deaths due to this cancer worldwide [1], while in 2018, there were around 570,000 cases and 311,000 deaths [4]. Most countries around the world have observed a decrease in cervical cancer incidence and mortality [1,4]; however, in many low-income and middle-income countries, the opposite trend has been observed [4,5,6,7,8,9,10]. The downward trend seen in most countries is largely attributed to a reduced risk of long-term high-risk human papillomavirus (hrHPV) infection, due to factors such as improved genital hygiene, reduced childbirth rates, a decrease in sexually transmitted infections, the implementation of screening programs, and hrHPV vaccinations [1,2,10]. Given these factors, the increasing trend observed in some countries is particularly concerning, as this cancer is considered a preventable disease [11].

The primary risk factor for cervical cancer is persistent hrHPV infection, associated with approximately 99.7% of cases [12,13,14]. Of the 20 HPV types linked to cervical cancer, types 16 and 18 account for about 75% of cases [2,14,15,16]. High-risk HPV infection can result in viral DNA integrating into cervical cell genomes, leading to the expression of E6 and E7 oncogenes, which disrupt the cell cycle by degrading the tumor suppressor proteins p53 and pRb [2,17]. Unlike retroviruses, HPV DNA integration is random, and oncogene expression requires an active viral promoter and/or a functional polyadenylation signal, which are not always present [17,18,19,20].

A meta-analysis by Bowden et al. [12] identified multiple cervical cancer risk factors beyond hrHPV infection. HIV infection appears to be a significant risk factor, increasing the likelihood of cervical cancer by at least six times [3]. Immunosuppression—mainly manifesting as a decrease in CD4+ T lymphocytes, which play a key role in eliminating HPV from the human body—plays a central role in this process [21,22]. Reports have also indicated associations between cervical cancer and both immunosuppressive treatment and autoimmune diseases (IBD, SLE, and RA), although these associations are not yet fully convincing [12,23,24]. Changes in the vaginal microbiome, including the depletion of Lactobacillus spp. and infection with Chlamydia trachomatis or Trichomonas spp., significantly increase the risk of hrHPV infection and the likelihood of cancer development [12,25,26,27,28]. Other significant risk factors mentioned for this cancer include smoking, a high number of sexual partners, medium- to long-term COCP usage, high parity, earlier age at first pregnancy, low vegetable intake, and increased vitamin C or selenium intake [12].

Effective primary prevention of cervical cancer involves hrHPV vaccinations, while secondary prevention is based on regular screening [29]. The secondary screening method uses regular cervical cytology, with liquid-based cytology, developed in recent years, emerging as the preferred method due to its advantages, including cost-effectiveness, fewer non-diagnostic smears, and the ability to test for hrHPV in the same sample [30,31,32]. Cervical cancer screenings are primarily conducted in high-income countries, with wide variation in participation rates among women [33]. According to data from Bruni et al. [33], only 69% of countries had a cervical cancer prevention program in place in 2021, and as many as two-thirds of women aged 30–49 had never participated in such a program. Vaccinating at least 90% of the population against hrHPV could significantly reduce cervical cancer incidence [34]. Unfortunately, such vaccination rates are achieved in only a few countries, with global coverage estimated at about 12% in 2018 [34,35].

Considering the above information, it is evident that high participation in prevention programs and a high vaccination rate against hrHPV are crucial for reducing cervical cancer incidence and mortality. Both state policies and women’s knowledge about this cancer play a central role in this process. Research on women’s knowledge of cervical cancer risk factors is, therefore, essential, as it enables the implementation of targeted educational programs in the future.

The objective of this study was to assess the knowledge of the surveyed women regarding cervical cancer prevention. The focus was on their understanding of risk factors for this cancer, their participation in screening (including frequency), and the prevalence of HPV vaccination in the studied population.

## 2. Materials and Methods

### 2.1. Study Design

The research method was a diagnostic survey in which a questionnaire was used. The study was conducted using a proprietary, anonymous online questionnaire available on the Google Cloud Platform from 2 January 2021 to 28 February 2021. Women received invitations to participate in the study, including a link to the study, via online groups of the Medical University of Lublin, as well as social media.

The authors’ self-designed survey questions were developed on the basis of the existing literature. The questionnaire was pilot-tested with a small sample group to identify any ambiguities or unclear items. After conducting the pilot study, the self-designed questionnaire was modified and evaluated. The research included women residing permanently in Poland, who were over 18 years of age and who consented to participate in the study. These factors were the criteria for inclusion in the anonymous study. The exclusion criteria included a lack of consent to participate in the study. All respondents were informed about the purpose and the anonymity of the research, and gave their consent.

### 2.2. Ethical Issues

The study was conducted in accordance with the human research principles of the Helsinki Declaration. The study protocol was approved by the Thesis Committee of the Medical University of Lublin on 25/11/2019. The Council of the Faculty of Health Sciences of the Medical University of Lublin approved the study (document no. 1/2019/2020) on 26/11/2019.

### 2.3. Questionnaire

The authors (A.D. and P.F.) designed and developed a questionnaire comprising 19 primary questions and 5 metric questions. The questionnaire was prepared according to the survey method for analyses of the relationships between variables. In order to obtain research material, a diagnostic survey method was used. The first part of the questionnaire included 5 closed-ended questions with one choice concerning sociodemographic variables like age, marital status, place of residence, education, material situation. The second part of the questionnaire constituted closed-ended questions with one or multiple choices. At the beginning of the questionnaire, respondents were provided with a written statement about the study’s purpose and were instructed on how to complete the survey, ensuring its full anonymity.

### 2.4. Statistical Analysis

The responses obtained from the respondents were analyzed descriptively. Charts were created using Microsoft Excel 2010, while statistical analyses were performed using the StatSoft Inc. STATISTICA (StatSoft, Cracow, Poland) data analysis software system, version 13.0, www.statsoft.com. Qualitative variables were presented as counts and percentages. To assess the dependence, strength, and direction of the relationships between variables, Pearson’s chi-square test was used. A statistical significance level of *p* < 0.05 was applied in all calculations.

## 3. Results

The study included 200 women from Poland, all of whom completed the questionnaire correctly. Over half of the participants (62.00%) were aged 18–25, and the majority (70.00%) lived in a town or city. Most respondents were single (51.50%), though a slightly smaller proportion (48.00%) reported being in a domestic partnership or marriage. Nearly all participants had completed secondary education (31.00%) or higher education (understood as at least a bachelor’s degree, 66.50%). The respondents were nearly evenly divided between students (49.50%) and working professionals (45.00%). Table 1 shows the characteristics of the study group.

**Figure 1:**
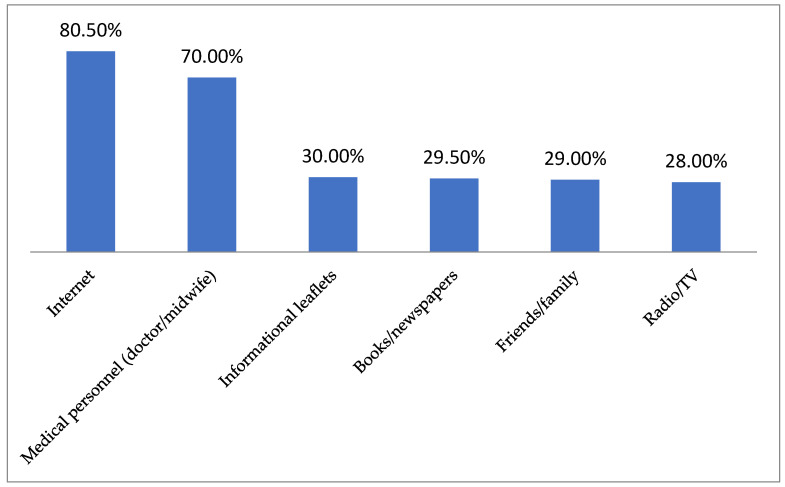
Sources of respondents’ knowledge about cervical cancer and its related preventive programs (multiple-choice question).

The respondents’ primary sources of knowledge about cervical cancer and its related preventive programs were the Internet and medical personnel (Figure 1).

Nearly half of the respondents visit a gynecologist (the purpose of such a visit may be to perform a Pap smear, although this is not necessary) at least once a year, while about one in five attend every six months on average. Similar percentages (almost every tenth participant) visit this specialist once every few years, only when concerning symptoms appear, or not at all. With increases in the age group, the percentage of women who visited a gynecologist at least once a year significantly increased, while the percentage of those who did not attend at all decreased, as shown in Table 2.

**Figure 2:**
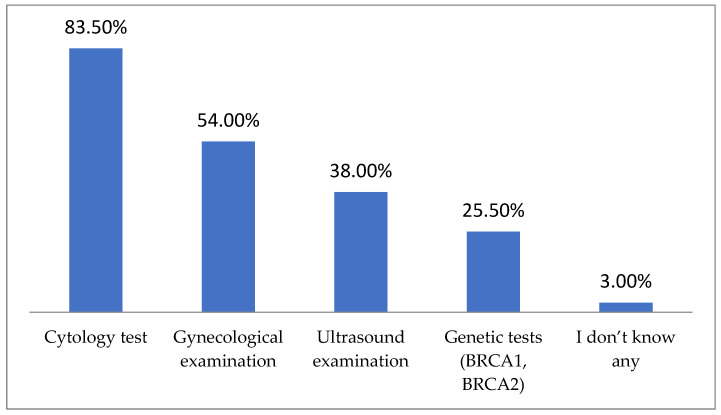
Cervical cancer prevention methods indicated by respondents (multiple-choice question).

The cytology test was the most commonly indicated cervical cancer prevention method among the respondents. Over half (54.00%) also pointed to gynecological examination (which should be understood as a pelvic examination), and one-third mentioned ultrasound examination (Figure 2).

Most respondents had their first cytology test after age 21, or within three years of becoming sexually active. One in four respondents had never undergone this test, while the remaining portion had it for the first time during pregnancy. The percentage of women who had never had a cytology test significantly decreased with age (37.10–9.30–0.00%, *p* < 0.0001), while younger women more often linked the timing of the test to the start of sexual activity (38.71% of women aged 18–25, 48.84% of women aged 26–35, and 21.21% of women aged 36–45, *p* < 0.0001). The recommendation of the Polish Ministry of Health is to perform the first cytology test at the age of 20–25 or shortly after the onset of sexual activity.

**Figure 3:**
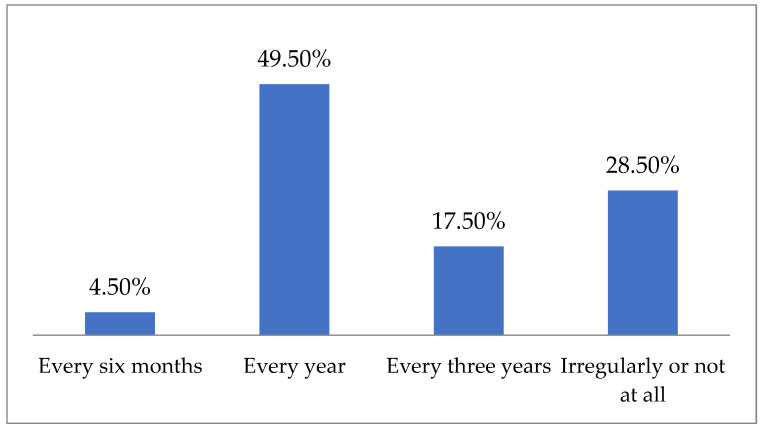
Frequency of respondents undergoing a cytology test.

Nearly half of the respondents undergo the test at regular annual intervals (Figure 3), and the percentage of those who have it irregularly or not at all decreases with age (44.35–16.28–12.12%, *p* < 0.0001). According to the majority of respondents, the first cytology test should be performed after the onset of sexual activity, with nearly one in five believing it should start at age 21, and the rest stating it should start after age 25.

More than half of the respondents (55%) were aware that mutations in the BRCA1 or BRCA2 genes predispose women to cervical cancer and breast cancer. Fewer than one in ten respondents associated these mutations only with cervical cancer (8.5%) or with breast and ovarian cancer (5%). The remaining respondents did not link these mutations to any type of cancer. The percentage of respondents associating BRCA1 and BRCA2 mutations with breast and ovarian cancer increased with age group (3.23–6.98–9.09%, *p* < 0.0108). Almost all respondents (96.5%) had not undergone genetic testing for BRCA1 and BRCA2 mutations.

Most respondents believed that the Polish Population-Based Early Detection Program for Cervical Cancer is available for individuals aged 25–59 (38.5%) or 25–60 (29.5%). Nearly one in five (21.5%) indicated the start of sexual activity as the eligibility criterion, while a small percentage of respondents mentioned the ages of 21–24 (3.5%) or were unable to choose an answer.

**Figure 4:**
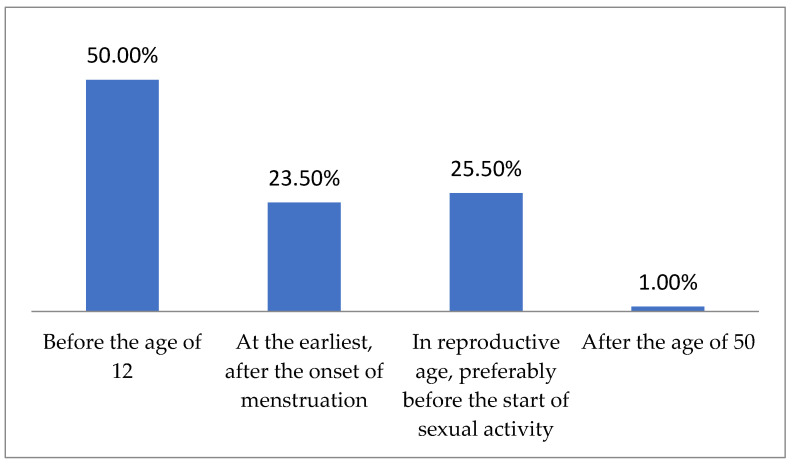
The ideal time for HPV vaccination, according to respondents.

When asked what plays a key role in the development of cervical cancer (HPV infection vs. genetic factors), most respondents pointed to HPV infection (81.50%), though paradoxically, the majority (86%) had not been vaccinated against this virus. The frequency of HPV infection being identified as the primary risk factor for cervical cancer increased with the respondents’ education level (20% for vocational, 80.65% for secondary, and 84.21% for higher, *p* < 0.0013). Nearly all respondents (99%) identified sexual contact as the primary route of HPV transmission. A significant majority also pointed to barrier contraception as the best method of prevention (88.5%), with a few respondents mentioning hormonal contraception (1.5%), avoiding intercourse during menstruation (4%), or delaying the start of sexual activity (6%). Barrier contraception was most frequently indicated by women in relationships (*p* < 0.0001), with the response rate increasing alongside the respondents’ education level (60% for vocational, 87.1% for secondary, and 90.23% for higher, *p* < 0.0004). When asked about the most effective method of HPV prevention (vaccination vs. condom use), the majority again indicated condom use (54.5%). The respondents were asked about the optimal age for receiving the hrHPV vaccine. Half of them indicated that it should be before the age of 12, while just over a quarter (25.50%) associated this period more with the onset of sexual activity than a specific age. Responses regarding the ideal time for HPV vaccination are shown in Figure 4.

Most respondents did not consider childbirth (53.5%) or breastfeeding (44.5%) as factors that reduce the risk of cervical cancer, with similar percentages unable to answer (23.5% and 27.5%, respectively). The majority of respondents reported no family history of cervical cancer (83%), while a small percentage (7.5%) indicated its presence, and nearly one in ten (9.5%) had no knowledge on this subject. The most frequently indicated symptom potentially associated with cervical cancer was vaginal bleeding (46.5%), with its indication frequency increasing with the respondents’ age group, as shown in Table 3.

## 4. Discussion

The conducted study shows that the respondents’ level of knowledge on cervical cancer risk factors and prevention remains generally good, especially concerning HPV’s association with cervical cancer. However, theoretical knowledge does not fully translate into regular gynecological visits, participation in the national cervical cancer prevention program, or, most importantly, HPV vaccination.

Respondents’ strong understanding of HPV’s role in cervical cancer development and methods to prevent HPV infection contrasts with studies conducted in many other countries, including Spain [36], Bangladesh [37], Zanzibar [38], India [39], and New Zealand [40], where knowledge on this topic remains very low. Questions regarding HPV’s role and the prevention of its infection were a crucial part of the survey, as this factor plays a key role in the development of nearly all cervical cancer cases [12,13,14]. It is noteworthy that respondents indicated barrier contraception methods as a more effective method of protection against HPV infection than vaccination, highlighting the need for broader awareness of this topic. Condom use provides good protection against HPV infection [41,42,43,44]; however, it is not 100% effective and certainly falls short of vaccination’s effectiveness [45,46,47,48,49]. In the past, reports have suggested that HPV vaccination might increase the frequency of risky sexual behaviors, but no scientific basis was found to support this correlation [50,51].

In the case of the conducted study, only 14% of the respondents were vaccinated against HPV, a particularly unsatisfactory result. Since 2023, HPV vaccination has been free in Poland for children and adolescents aged 9–18, yet its popularity remains low, with its population vaccination coverage estimated at around 10–20%, roughly corresponding to the study’s results [52,53]. The HPV vaccination program was introduced relatively late in Poland compared to other EU countries, many of which implemented such programs before 2010 [53]. Nonetheless, HPV vaccination coverage in EU countries varies significantly, from around 20% in France to as high as 80–90% in Hungary and Belgium [53]. The global HPV vaccination rate is estimated at around 12%, but this rate varies greatly depending on the region and is generally higher in wealthier countries [35]. The World Health Organization (WHO) Global Strategy for the Elimination of Cervical Cancer recommends that every country should reach the 90–70–90 targets by 2030, meaning that 90% of girls should be fully vaccinated with the HPV vaccine before age 15, 70% of women should undergo screening before age 35 (and again before age 45), and 90% of diagnosed women should be undergoing treatment (both those with precancerous lesions and those with advanced disease) [54]. According to WHO models, achieving these targets would reduce cervical cancer cases by 42% by 2045 and by 97% by 2120; however, given the data above, global realization seems unlikely [54].

Mutations in the BRCA1 and BRCA2 genes are known and significant risk factors for breast and ovarian cancer [55,56]. A meta-analysis by Lee et al. [56] linked these gene mutations to an increased risk of pancreatic cancer and uterine cancer, but found no association with brain cancer, colorectal cancer, prostate cancer, bladder and kidney cancer, cervical cancer, or malignant melanoma. Due to the fact that information about mutations in the BRCA1 and BRCA2 genes acting as risk factors for breast cancer appears in various informational campaigns in Poland, the authors decided to check whether the respondents associate these mutations with the risk of developing the corresponding cancer. Unfortunately, only a few individuals in the study group provided the correct answer, which indicates the need for further education in this area.

A high number of births is a confirmed risk factor for cervical cancer [12,57]; however, the cause of this phenomenon is not well understood. It is suspected that migration of the endocervix during pregnancy [58,59] and trauma to the uterine cervix during vaginal delivery [57,60] play a crucial role. The link between breastfeeding and cervical cancer risk remains unclear [61,62]. Jin et al. [61] found that the risk of cervical cancer increases with breastfeeding periods shorter than 13 months compared to longer durations, but this association was not confirmed in another study [62]. Knowledge of the relationship between these factors and cervical cancer is unsatisfactory among the studied population.

The WHO recommends a cervical cancer screening program for women aged 30–50 years using hrHPV DNA testing every 5–10 years or, if this is not feasible, cytology tests every 3 years [63]. The European Commission recommends a cervical cancer screening program for women aged 30–65 using hrHPV DNA testing every 5 years or more [64]. In Poland, the cervical cancer prevention program covers women aged 25–64 (up to 2023, it was 25–59 years) and funds a cytology test every 3 years, with those affected by significant risk factors (e.g., HIV infection) able to take this test annually [65]. Given the information above, it should be noted that the Polish prevention program covers a broader age group than those recommended by the WHO and the European Commission; however, it does not include hrHPV DNA tests for widespread use. Most respondents in this study were able to identify the approximate target age group for the prevention program.

Regarding the available guidelines, the optimal time for the first cervical cancer screening is indicated to be age 21 [66] or 25 [63,64], regardless of sexual activity. In this study, respondents associated the start of cervical cancer screening participation mainly with the initiation of sexual activity, indicating a need to increase their knowledge on this topic. Although nearly one in four respondents in this study had not yet participated in a preventive cytology test, this phenomenon does not appear particularly concerning, as the vast majority were within the 18–25 age group, which, based on the cited guidelines and the national program, does not require such screening [63,64,65] or should only include part of this group [66]. It is also noteworthy that nearly half of the study group undergoes preventive cytology annually, even more frequently than recommended.

Slightly less than three-quarters of the respondents from the conducted study visit a gynecologist once a year or more often. Annual gynecological examinations are considered by many specialists in this field to be extremely important preventive actions, also receiving positive patient feedback [67,68,69,70,71]. However, opinions differ on the superiority of annual gynecological exams over exams conducted every few years (if a cervical cancer prevention program is in place)—some organizations recommend annual exams (e.g., the American College of Obstetricians and Gynecologists) [67], while others recommend less frequent visits (e.g., the American College of Physicians) [67,71].

Cervical cancer usually has no symptoms in its early stages, with more noticeable symptoms—such as inter-menstrual bleeding, post-menopausal bleeding, and offensive vaginal discharge—typically appearing only at an advanced stage, highlighting the importance of early detection programs [2,72]. Respondents from the conducted study demonstrated good knowledge of possible cervical cancer symptoms, which also aligns with findings from other studies [72].

The primary sources of the respondents’ knowledge about cervical cancer and its associated prevention programs were the internet and medical personnel. In most studies on this topic, medical personnel remain the primary source of knowledge for respondents [72,73,74,75,76,77,78], although in some cases, mass media (including the internet) also predominate [79,80,81]. While medical personnel remain the optimal source of health information, mass media can reach a larger audience and, thus, contribute to greater public awareness of a given topic [72]. Ensuring the substantive quality of information transmitted through mass media is, therefore, a key issue.

Despite the relatively optimistic conclusions drawn from the conducted study, it should be noted that cervical cancer is a significant public health problem in Poland [82]. The incidence rate of this cancer in Poland is significantly higher than in most European countries, and the mortality rate is approximately twice the European average [82]. The causes of this situation primarily include the low HPV vaccination rate and the relatively low popularity of screening in the population at a national level [82]. It should be noted that the HPV vaccination program was only recently introduced in Poland, meaning that this situation may change in the coming years. The results should be interpreted with caution due to the small study group. A subsequent survey employing a larger sample size would be beneficial.

## 5. Study Limitations

Despite the study’s strengths, which include the concise collection of necessary information and the inclusion of questions addressing various aspects of cervical cancer prevention, it is essential to consider its limitations, among which the limited size of the study group seems critical. It should also be noted that an open-ended questionnaire might have provided more valuable information; however, gathering an adequate number of respondents in such a case could have been challenging.

## 6. Conclusions

The level of knowledge and the participation of the surveyed women in cervical cancer screening remains at a satisfactory level in Poland. Despite relatively good knowledge about the role of HPV in cervical cancer development, the popularity of vaccinations against this virus remains relatively low in Poland. Some socio-demographic factors, such as age and education, significantly differentiated the knowledge and health attitudes of the respondents. It is necessary to create appropriate educational programs to increase women’s knowledge on selected aspects of cervical cancer prevention, to encourage more people to participate in screening tests, and, most importantly, to increase the population’s vaccination rate against HPV. The research sample was relatively small and, therefore, a representative sample may not be guaranteed, which limits the generalizability of the study results.

## Figures and Tables

**Table 1:** The demographic characteristics of the study group.

Study Group (n = 200)	
Age (years)	
18–25	124 (62.00%)
26–35	42 (21.00%)
36–45	34 (17.00%)
Permanent residence	
Village	60 (30.00%)
Town/city	140 (70.00%)
Marital status	
Single	103 (51.50%)
Informal/marital relationship	96 (48.00%)
Widow	1 (0.50%)
Education	
Completed primary school	0 (0.00%)
Completed vocational school	5 (2.50%)
Completed secondary education	62 (31.00%)
Completed higher education	133 (66.50%)
Main occupation	
Student	99 (49.50%)
Unemployed	6 (3.00%)
Working professional	90 (45.00%)
Household	5 (2.50%)

n, number.

**Table 2:** Frequency of gynecologist visits vs. age of respondents.

Frequency of Gynecologist Visits vs. Age of Respondents						
Age Range	Once a Year n (%)	Every Six Months n (%)	Every Few Years n (%)	I Don’t Go at All n (%)	Only When Concerning Symptoms Appear n (%)	Row Total n
18–25 years	46 (37.10%)	27 (21.77%)	9 (7.26%)	16 (12.90%)	26 (20.97%)	124
26–35 years	23 (53.49%)	9 (20.93%)	6 (13.95%)	2 (4.65%)	3 (6.98%)	43
36–45 years	21 (63.64%)	6 (18.18%)	6 18.18%	0 (0.00%)	0 (0.00%)	33
Total	90	42	21	18	29	200
Pearson’s chi-square: 24.71460, df = 8, *p* < 0.0017						

n, number; %, percent.

**Table 3:** The most common symptom of cervical cancer according to respondents vs. ages of the respondents.

The Most Common Symptom of Cervical Cancer According to Respondents					
Age Range	Vaginal Bleeding n (%)	Pain During Intercourse n (%)	Discharge n (%)	Irregular Menstrual Bleeding n (%)	Row Total n
18–25 years	54 (43.55%)	25 (20.16%)	18 (14.52%)	27 (21.77%)	124
26–35 years	19 (44.19%)	8 (18.60%)	7 (16.28%)	9 (20.93%)	43
36–45 years	20 (60.61%)	2 (6.06%)	5 (15.15%)	6 (18.18%)	33
Total	93	35	30	42	200
Pearson’s chi-square: 4.989188, df = 6, *p* < 0.005					

n, number; %, percent.

## Data Availability

Data are contained within the article.
